# Printable and Antiferromagnetic Mn(OH)_2_@Te–O Core–Shell Nanosheets

**DOI:** 10.1021/acs.chemmater.5c02655

**Published:** 2026-01-27

**Authors:** Fang Yuan, Jiaze Xie, Ratnadwip Singha, Christie S. Koay, Sigalit Aharon, Guangming Cheng, Brianna L. Hoff, Vojtech Kundrat, Xiaoyu Song, Sudipta Chatterjee, Lothar Houben, Jakub Zalesak, Nan Yao, Leslie M. Schoop

**Affiliations:** † Department of Chemistry, Princeton University, Princeton, New Jersey 08544, United States; ‡ Department of Physics, 6740 Indian Institute of Technology Guwahati, Assam 781039, India; § Princeton Materials Institute, 28678 Princeton University, Princeton, New Jersey 08544, United States; ∥ Department of Chemistry, Faculty of Science, Masaryk University, Kamenice 5, Brno 62500, Czechia; ⊥ Department of Chemistry, Princeton University, Princeton, New Jersey 08544, United States; # Department of Chemical Research Support, Weizmann Institute of Science, Rehovot 7610001, Israel; ∇ Chemistry and Physics of Materials, University of Salzburg, 5020 Salzburg, Austria

## Abstract

Core–shell nanomaterials provide a versatile platform for tuning physical properties and integrating complementary functionalities in nanoscale systems, but their synthesis often requires multistep procedures and precise control over composition, morphology, and interfaces. Achieving core–shell architectures in nanosheets is particularly challenging due to the difficulty of controlling growth direction and interfacial formation. Here, we describe a one-step chemical exfoliation process that produces core–shell nanosheets from the highly air-sensitive compound Li_1+*x*_MnTe_2_. Brief sonication in Milli-Q water under ambient conditions yields a dark gray suspension of nanosheets within 10 min, which remains stable in air for at least 31 days. Powder X-ray diffraction, scanning electron microscopy with energy-dispersive X-ray spectroscopy, inductively coupled plasma–optical emission spectrometry, and transmission electron microscopy indicate the formation of few-layer crystalline Mn­(OH)_2_ cores encapsulated by amorphous Te–O shells. Magnetic measurements show antiferromagnetic ordering in the restacked nanosheets. The suspension can be readily deposited onto coated glass, polyethylene terephthalate, and Si/SiO_2_ substrates to form uniform films, with electrical transport measurements indicating resistances on the order of megaohms at room temperature. These results demonstrate chemical exfoliation as an effective approach for producing core–shell nanosheets with magnetic and electronic functionality.

## Graphical abstract

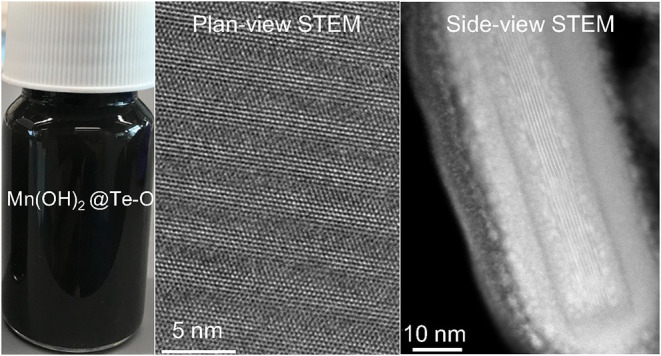

## Introduction

Core–shell nanomaterials represent an important subclass of composite nanostructures in which a central core is surrounded by one or more shells of different compositions and physicochemical properties. This architecture enables the integration of complementary functionalities. By carefully selecting core and shell components, properties such as optical absorption, magnetic response, surface reactivity, and thermal stability can be finely tuned.^1–7^ Therefore, core–shell nanomaterials have been widely explored for applications in catalysis, energy storage, biomedical imaging, drug delivery, and electronic devices.^8–11^ To meet the increasing demand, a variety of synthetic strategies have been established, including seed-mediated growth, sol–gel methods, coprecipitation, hydrothermal/solvothermal synthesis, atomic layer deposition, and epitaxial assembly.^1,12–16^ Despite these advances, the synthesis of core–shell nanostructures remains inherently complex, as it is usually a multistep process and also requires precise control over composition, morphology, and interfacial chemistry. Shell formation is particularly critical and often depends on precursor deposition onto the core surface, where parameters such as the precursor concentration, temperature, pH, redox potential, and surfactant environment must be carefully optimized to achieve uniform coverage. In addition, shell thickness must be carefully controlled: insufficient coverage leaves the core vulnerable to degradation, whereas overly thick shells can suppress key functionalities of the core, such as fluorescence or catalytic activity.^17^ Maintaining the structural integrity of the core and shell components under reaction conditions, particularly at elevated temperatures, is critical to ensure the functionality of core–shell nanomaterials. In this situation, synthesis strategies that operate under ambient conditions are especially attractive, as they avoid thermal degradation while enabling stable core–shell architectures to form at room temperature.

The unique architecture of core–shell nanomaterials also provides a potential strategy to stabilize air-sensitive compounds. For example, encapsulating easily oxidized cores with air-stable noble shells can effectively prevent degradation.^11^ Manganese­(II) hydroxide (Mn­(OH)_2_) is one such material of interest. Currently, it is widely used as an intermediate for producing MnO_2_ ^18^ and has versatile applications due to the redox and adsorptive performance of its oxidized Mn phases. For example, Mn­(OH)_2_ has been widely investigated as a cathode material or precursor in rechargeable alkaline and lithium-ion batteries due to its redox activity.^19–22^ These works often involve the electrochemical transformation from layered MnO_2_ to Mn­(OH)_2_ and back. In water treatment, the oxidized phases of Mn­(OH)_2_ can remove contaminants such as arsenic and heavy metals through adsorption and redox reactions.^23–25^ Furthermore, the incorporation of a small amount of Mn­(OH)_2_ into Co­(OH)_2_/Ni­(OH)_2_ nanorods significantly enhanced their electrochemical performance through a surface-charge–controlled storage mechanism, demonstrating that mixed metal hydroxides are promising electrode materials for supercapacitor applications.^26^ Beyond these applications based on its redox activity, the intrinsic structure and physical properties of Mn­(OH)_2_, especially its two-dimensional (2D) counterparts, remain underexplored, largely because the compound is highly air-sensitive. We found only one paper showing that the bulk Mn­(OH)_2_ is antiferromagnetic below 12 K.^27^ However, the magnetic properties of its 2D counterparts have not yet been investigated. Pure Mn­(OH)_2_ is pale pink or white, but after air exposure, Mn^2+^ ions rapidly oxidize to higher oxidation states such as Mn^3+^ or Mn^4+^, producing brown or black oxides. This transformation can occur in minutes, necessitating storage in an inert atmosphere or in deoxygenated water. Designing core–shell architectures with Mn­(OH)_2_ as the core provides a pathway to improve its stability and expand its applications. Furthermore, ultrathin 2D-layered metal hydroxides have recently emerged as promising platforms for catalysis, energy conversion, and energy storage.^28–30^ Scalable synthesis methods for Mn­(OH)_2_ nanosheets, such as chemical exfoliation, may further enhance their catalytic activity. Combining core–shell strategies with chemical exfoliation thus provides a way to transform air-sensitive Mn­(OH)_2_ into colloidally stable, printable nanomaterials in liquid media under ambient conditions.

Although numerous synthetic strategies have been developed to produce core–shell nanomaterials, a simple one-step synthesis routine remains challenging. Moreover, when it comes to nanosheets, achieving a well-defined core–shell architecture is particularly difficult. Unlike bulk or nanoparticle systems, where growth can be guided more uniformly, 2D nanosheets present unique synthesis difficulties due to their highly anisotropic nature. Controlling the growth direction is already challenging on its own, and introducing an additional shell layer without disrupting the integrity of the core adds a further level of complexity.^31^ These constraints make the design of core–shell nanosheets an especially demanding and scientifically important goal. In this study, we explore quasi-layered and highly air-sensitive LiMnTe_2_ as a precursor for the formation of core–shell nanosheets. As we found a higher Li concentration in all samples, we refer to our samples as Li_1+*x*_MnTe_2_. LiMnTe_2_, as reported in the literature, crystallizes in a trigonal structure, where lithium ions occupy interlayer sites alternating with edge-sharing MnTe_6_ octahedral layers.^32^ Such quasi-layered compounds with intercalated Li^+^ have strong solvation ability in aqueous solutions, which often facilitates exfoliation due to weakened interlayer interactions arising from expanded interlayer spacing.^33–37^ These structural characteristics suggest that LiMnTe_2_ could be a promising precursor for 2D nanostructures. However, to date, no study has explored its use in this direction yet. The primary obstacle is its intrinsic instability, which poses substantial synthetic and handling challenges. Importantly, the combination of intercalated lithium ions and redox-active manganese ions, which can adopt multiple oxidation states, makes LiMnTe_2_ an attractive candidate for the production of 2D materials through air exposure and redox reactions.

In this work, for the first time, we report the synthesis of core–shell nanosheets consisting of few-layer Mn­(OH)_2_ cores encapsulated by an amorphous Te–O shell. The formation mechanism was investigated by linking the chemical exfoliation and redox processes of Li_1+*x*_MnTe_2_ in Milli-Q water under ambient conditions. Unlike most core–shell nanomaterials that require multistep synthesis under strict thermal control, we demonstrate an in situ one-step approach that produces core–shell nanosheets under ambient conditions, producing large quantities after only 10 min of sonication in Milli-Q water. The structure and composition of the resulting nanosheets were characterized by using powder X-ray diffraction (PXRD), transmission electron microscopy (TEM), and energy-dispersive X-ray spectroscopy (EDS). Finally, we examined the physical properties of the nanosheets and correlated them with their structural features. In general, this study presents a scalable one-step synthesis of air-stable core–shell nanosheets with multifunctional properties, including antiferromagnetism and printability potential for electronic and optoelectronic applications. It is interesting to apply this method to other unstable quasi-layered materials to form additional core–shell magnetic nanosheets.

## Experimental Section

### Synthesis

Stoichiometric amounts of Mn and Te were combined with a 50% excess of Li in an Al_2_O_3_ crucible. The mixture was annealed in sealed evacuated quartz tubes, following the heat profile described by Kim et al.^32^ This established synthesis method consistently yielded single ruby-red Li_1+*x*_MnTe_2_ crystals. The parent Li_1+*x*_MnTe_2_ crystals were kept in an Ar-filled glovebox. Subsequently, the Li_1+*x*_MnTe_2_ crystals were brought to air in an enclosed glassy vial and subjected to sonication in Milli-Q water under ambient conditions at a solid-to-liquid ratio of 1 mg:4 mL using a Branson 1800 sonicator in high-power mode for 10 min. Following sonication, the liquid was carefully transferred to a centrifuge tube and centrifuged at 2000 revolution per min (rpm) at 4 °C for 20 min. The resulting supernatant, which is rich with thin nanosheets, was collected for further characterization, while sediments containing unexfoliated residue were discarded. In this way, a colloidally stable nanosheet ink can be obtained in Milli-Q water, with a yield of approximately 15% after subtracting unexfoliated sediments. For structural and physical property measurements, the upper nanosheet suspension was subjected to an additional centrifugation step at 12,000 rpm at 4 °C for 1 h to facilitate the restacking of the nanosheets at the bottom of the centrifuge tube. The collected nanosheets were finally dried under vacuum at room temperature.

### Scanning Electron Microscopy

The sample morphology was captured using a Helios DualBeam focused ion beam (FIB)/scanning electron microscope (SEM) outfitted with EDS. Nanosheet suspension was transferred and dried on a Si/SiO_2_ wafer. Li_1+*x*_MnTe_2_ bulk crystals and restacked nanosheets on the Si/SiO_2_ wafer were mounted on carbon tape secured to aluminum sample holders before being transferred to the SEM chamber.

### Inductively Coupled Plasma-Optical Emission Spectrometry

Li content was quantitatively measured using inductively coupled plasma-optical emission spectrometry (ICP-OES) with an Agilent 5800 instrument. Before analysis, the samples were digested in concentrated acid using a CEM Discover SP-D 80 Microwave Digester and subsequently diluted in Milli-Q water. 5 mg of parent Li_1+*x*_MnTe_2_ and restacked nanosheets were digested in 50 mL of aqueous solution. The resulting aqueous solution was then introduced into the ICP-OES instrument through a nebulizer. For calibration, Li ICP standards were prepared at concentrations of 1, 5, 10, 20, and 50 ppm from a 1000 ppm commercial Li ICP solution in a similar matrix as the samples. Each standard was measured in triplicate, and the resulting intensity values were used to construct a calibration curve that was then applied to determine the Li concentrations of the samples.

### Powder X-ray Diffraction

PXRD was employed to determine the crystal structures and purities of the samples. PXRD measurements were performed on a STOE STADI PXRD instrument, operating in Debye–Scherrer geometry with monochromatic Mo-*K*_α1_ radiation (λ_*Kα*1_ = 0.7093Å) and a single Mythen detector. Finely ground samples were sealed in 0.5 mm quartz capillaries for analysis.

### TEM Sample Preparation

#### Cross-Sectional TEM Sample Preparation

Cross-sectional TEM samples were prepared by cutting the Si/SiO_2_ wafer containing restacked nanosheets and then thinned to a thickness of approximately 60 nm using Helios 5 FX FIB-SEM from Thermo Fisher Scientific. The TEM thin-lamellar samples were further polished using a 2 kV gallium ion beam to minimize surface damage caused by the high-energy focused ion beam. The resulting TEM lamella was then transferred onto a FIB lift-out TEM grid and promptly loaded into the high-vacuum chamber of TEM.

#### Plan-View TEM Sample Preparation

Plan-view TEM samples were prepared by depositing droplets of nanosheet suspension onto 400 mesh Cu TEM grids with a carbon lacey support film.

### Scanning/Transmission Electron Microscopy

The selected area electron diffraction (SAED) and TEM images were acquired using a Talos F200X scanning and transmission electron microscope (S/TEM) operating at an accelerating voltage of 200 kV. High-resolution transmission electron microscopy (HRTEM) images were obtained using a Titan Cubed Themis 300 double Cs-corrected S/TEM operating at 300 kV, with a spatial resolution of 0.07 nm and an energy resolution of 0.8 eV. Additionally, the Titan Cubed Themis S/TEM is equipped with a super-X EDS system for elemental mapping and a Gatan Quantum SE/963 P postcolumn energy filter for energy-filtered TEM and electron energy-loss spectra (EELS) data acquisition.

### Magnetic Property Measurement

Magnetic properties were assessed by using a Quantum Design SQUID-VSM magnetic property measurement system (MPMS3). The dried restacked Mn–Te–O nanosheets were mounted into plastic capsules for magnetic measurements.

### Electronic Transport Measurement

Electronic transport measurements were performed using the ETO option of a physical property measurement system (PPMS; Quantum Design). To prepare the device, a suspension containing the core–shell nanosheets was dropped onto a prepatterned Si/SiO_2_ wafer. The electrical connections were then made in a four-probe setup using silver paint and gold wires.

## Results and Discussion

### Chemical Exfoliation

Hydrophilic cations cause significant swelling of quasi-layered inorganic materials due to their absorption of H_2_O molecules in the interlayer spacing in aqueous solution.^37,38^ Among all alkaline metal ions, Li^+^ has the highest hydration enthalpy of −519 kJ/mol, suggesting the largest swelling ability, making it favorable for chemical exfoliation.^39^ Therefore, Li-intercalated transition metal dichalcogenides (TMDs) are easily hydrated in an aqueous solution. For example, Li-intercalated MoS_2_, LiMoS_2_, transforms into Li­(H_2_O)_*x*_MoS_2_, accompanied by significantly expanded interlayer spacing.^40,41^ To promote the chemical exfoliation of Li_1+*x*_MnTe_2_, we first attempted to weaken the bonding between layers by solvating Li^+^ in Milli-Q water. As a comparison, chemical exfoliation was done in different solvents, including ethanol, methanol, and acetone. However, only Milli-Q water provided a strong enough solvation (Figure S1). Although water, ethanol, and methanol are all protic solvents, water stands out due to its higher polarity, which is crucial for Li^+^ solvation of Li_1+*x*_MnTe_2_. We also tested acetone, a polar aprotic solvent that coordinates Li^+^ through its carbonyl oxygen. However, its solvation appears to be weaker than that of water, making it unable to break the electrostatic bond between the Li^+^ and the sandwiched [MnTe_2_]^–^ layers.

Figure 1 illustrates the exfoliation process of Li_1+*x*_MnTe_2_ in Milli-Q water. Upon exposure to air, Milli-Q water was added to the vial containing Li_1+*x*_MnTe_2_. The formation of gas bubbles indicates a redox reaction, in which protons are reduced to H_2_, which usually occurs in quasi-layered compounds with intercalated alkali ions.^42,43^ To further delaminate Li_1+*x*_MnTe_2_ into nanosheets, sonication was applied. Due to the high air sensitivity of Li_1+*x*_MnTe_2_, the redox reaction between Li_1+*x*_MnTe_2_ and water was highly vigorous under ambient conditions. Large amounts of gas bubbles were generated during sonication, accompanied by a change in the color of the liquid. Sonication, coupled with the force of gas bubbles generated from the redox reaction, facilitated the exfoliation of Li_1+*x*_MnTe_2_, resulting in a dark gray suspension in just 10 min of sonication. The generated suspension remains stable without agglomeration for up to 24 h.

**Figure 1:**
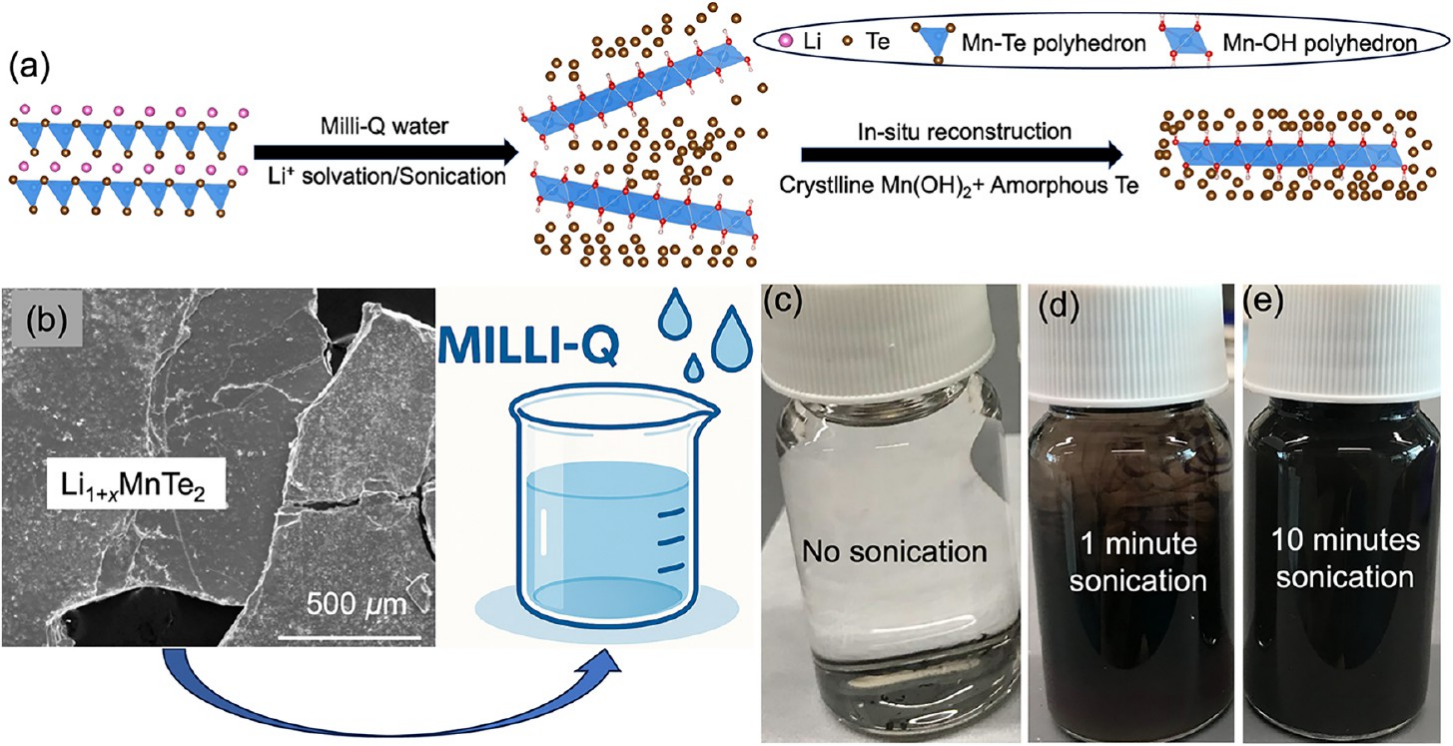
Schematic illustrating the synthesis process of Mn–Te–O core–shell nanosheets. (a) Schematic illustration of the synthesis of Mn–Te–O core–shell nanosheets. (b) Mixture of bulk Li_1+*x*_MnTe_2_ materials and Milli-Q water. (c) Li_1+*x*_MnTe_2_ mixed with Milli-Q water. (d) Mixture of Li_1+*x*_MnTe_2_ and Milli-Q water with 1 min sonication. (e) Dispersed Mn–Te–O nanosheets in Milli-Q water after 10 min sonication.

### Structure and Composition Characterizations of Restacked Nanosheets

To gain deeper insight into the chemical exfoliation process, it is essential to examine the structure and composition of the resulting nanosheets. Therefore, we performed structural and compositional characterizations on the dried, restacked material and compared it with the parent Li_1+*x*_MnTe_2_ crystals. Figure 2a shows that the PXRD of the parent compound agrees well with the reported trigonal *P*3*m* structure. As shown in Figure 2d, the restacked nanosheets adopt a structure markedly different from that of the parent Li_1+*x*_MnTe_2_, consisting of 91.1 at. % Mn­(OH)_2_, 7.1 at. % Te, and 1.8 at. % MnTe_2_. Additionally, a few satellite peaks were observed in the PXRD pattern of the restacked sample, such as the peak at 4.4° of 2θ, corresponding to a lattice spacing of ∼9.0 Å. This lattice spacing is much larger than that present in the phases included in the Rietveld refinement of restacked nanosheets. More details about the large lattice spacing are discussed in the TEM section below. SEM morphological analysis revealed that the parent crystal exhibits a layered structure, whereas the exfoliated sample consists of numerous restacked nanosheets with average lateral dimensions of several 100 nm to 1 μm, confirming the successful exfoliation of the parent Li_1+*x*_MnTe_2_ (Figure 2b,2e). EDS analysis further indicated that the parent compound has a Mn/Te ratio of 1:2, while the average composition of the collected nanosheets is close to MnTe (Figure 2c,f). Additionally, oxygen was detected in both parent and exfoliated samples, with a significant increase in the exfoliated material. This finding is in agreement with the PXRD results, which indicates the formation of the Mn­(OH)_2_ phase. To monitor the change in lithium concentration during exfoliation, Li quantification was performed by using ICP-OES. Table S1 summarizes the Li content of the parent compounds and the restacked core–shell nanosheets. Our results consistently show that the parent compound contains a significantly higher Li content than reported in the literature for LiMnTe_2_, whereas the restacked core–shell nanosheets exhibit negligible Li content. We note that postsynthetic treatments in solvents such as water, ethanol, pyridine, acetonitrile, ethylenediamine, dimethylformamide, and dimethyl sulfoxide can effectively deintercalate Li, as reported for air-sensitive LiMnBi.^44^ In fact, the original paper that reported the compound LiMnTe_2_ states that samples were washed with methanol to remove a Li_2_Te impurity.^32^ We believe that this washing step may have removed extra lithium, which would explain the discrepancy between our observed Li content and that reported in the literature. To verify this hypothesis, we washed our samples with methanol and observed a dramatic reduction of Li. After methanol washing, ICP-OES measurements performed on digested large single crystals suggest a composition close to LiMnTe_2_, in agreement with literature values. If we first crushed large crystals into small pieces, the digested products contained almost no Li after the same methanol washing. Additionally, PXRD results in Figure 2a suggest that our parent compound does not show a Li_2_Te impurity. Thus, we conclude that methanol washing does more than just remove impurities, which explains why we consistently find a higher Li content in our samples. These results confirm that lithium is completely removed in the nanosheets. Additionally, the structure of LiMnTe_2_ can accommodate excess Li; hence, we decided to name the starting material Li_1+*x*_MnTe_2_.

**Figure 2:**
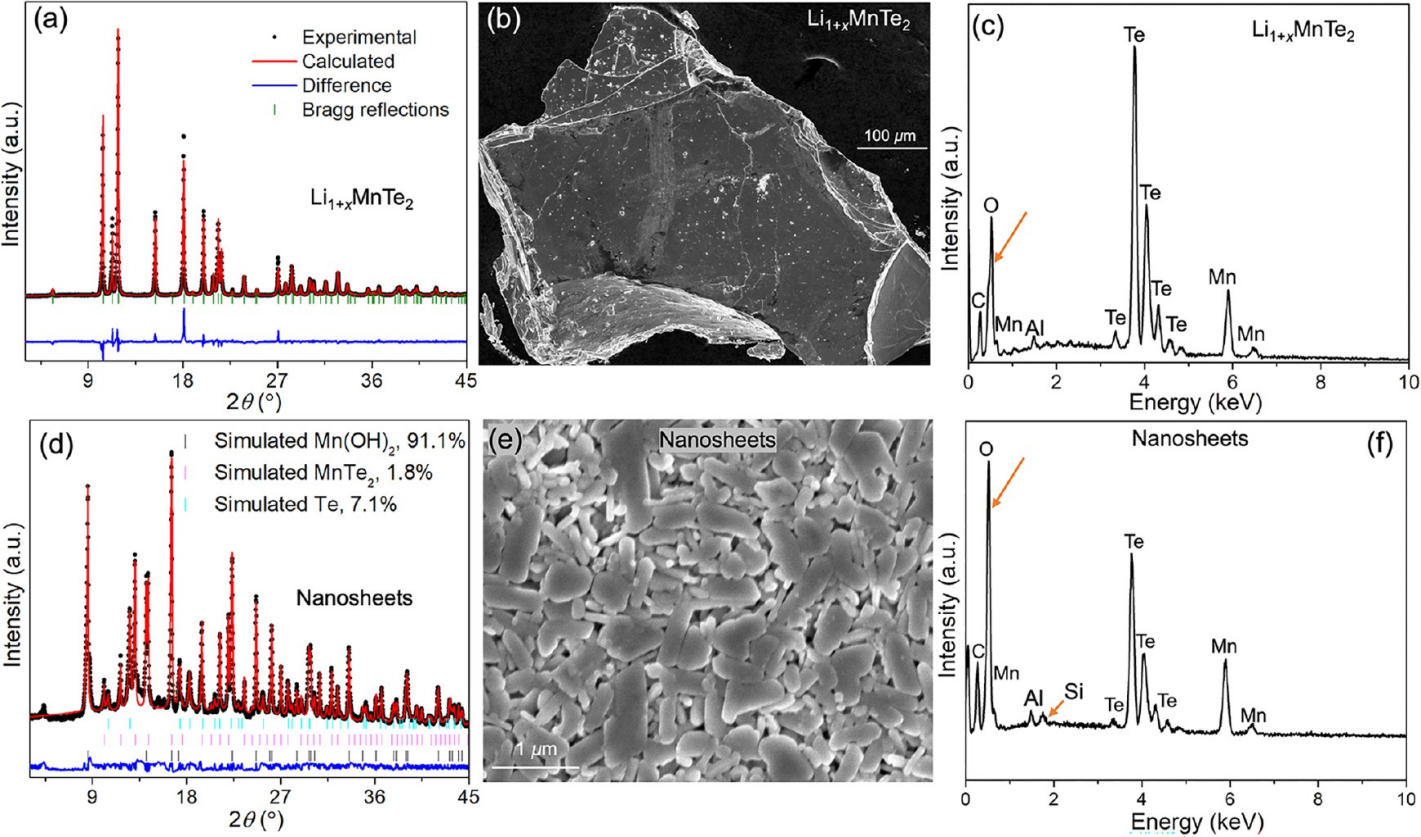
Structural and compositional characterizations. (a) PXRD pattern of parent Li_1+*x*_MnTe_2_, including the Rietveld refinement fit, difference curve, and Bragg peak positions. (b) SEM image of parent Li_1+*x*_MnTe_2_. (c) EDS spectrum of the bulk Li_1+*x*_MnTe_2_ compound. (d) PXRD pattern of restacked nanosheets with corresponding Rietveld refinement, difference curve, and Bragg peak positions. (e) SEM image of nanosheets, obtained by drop casting the nanosheet suspension on a Si/SiO_2_ wafer. (f) Corresponding EDS spectrum. The orange arrow highlights the sharp increase in the relative oxygen content of the nanosheets.

The structural and compositional characterizations suggest that two chemical reactions take place during chemical exfoliation, as summarized by eqs 1 and 2. The chemical reaction for the formation of Mn­(OH)_2_ and Te can be attributed to eqs 1. Li_1+*x*_MnTe_2_ reduces H_2_O to form hydrogen; at the same time, Li_1+*x*_MnTe_2_ decomposes due to the redox reaction, forming Mn­(OH)_2_, LiOH, and Te. Beyond the first chemical reaction, there is a secondary chemical reaction that generates MnTe_2_ and H_2_ as side products, as shown in eqs 2.

1 $$2{\mathrm{Li}}_{1+x}{\mathrm{MnTe}}_{2}+2\left(3+x\right)H_{2}O\to 2\mathrm{Mn}{\left(\mathrm{OH}\right)}_{2}+2\left(1+x\right)\mathrm{LiOH}+\left(3+x\right)H_{2}+4\mathrm{Te}$$

2 $$2{\mathrm{Li}}_{1+x}{\mathrm{MnTe}}_{2}+2\left(1+x\right)H_{2}O\to 2{\mathrm{MnTe}}_{2}+2\left(1+x\right)\mathrm{LiOH}+\left(1+x\right)H_{2}$$

While the composition analysis of Li, Mn, and O appears reasonable, the Te content in the restacked nanosheets is much higher than the values obtained from the Rietveld refinement. One possible reason is that some Te exists in amorphous phases and therefore cannot be detected by PXRD. Further details on the structural analysis of Te are provided in the cross-sectional STEM results. Additionally, determining the accurate composition of restacked nanosheets solely from SEM-EDS ratios is challenging because of the difficulty in preparing flat, homogeneous specimens and the presence of multiple phases with heterogeneous distributions. To overcome this technical limitation, we performed quantitative elemental mapping and composition analysis on individual nanosheets using STEM-EDS, which provides more reliable compositional data.

### Characterization of Exfoliated Nanosheets Using S/TEM and EELS

Figure 3 presents a TEM characterization of the exfoliated nanosheets. In the initial suspension, each droplet contained a large number of overlapping nanosheets, making it difficult to get HRTEM and SAED (Figure S2a). To address the issue, the suspension was diluted 20 times in Milli-Q water (Figure S2b). Dispersive nanosheets with lateral dimensions of up to several micrometers were observed (Figure S3). Notably, the nanosheets have excellent air stability. Air stability was evaluated by periodic TEM analysis of samples stored under ambient laboratory conditions. The core–shell nanosheets remained crystalline after 31 days of air exposure but showed clear degradation after 34 days, defining a well-resolved stability window. Pronounced changes in morphology and SAED patterns observed after 34 days indicate the onset of structural degradation (Figure S4). HRTEM images (Figure 3) reveal a complex but periodic plan-view structure. Consistent SAED patterns were observed across different nanosheets, displaying sharp diffraction spots indicative of good crystallinity (Figures S5 and 3c). Furthermore, SAED patterns suggest a structure comprising multiple phases. Table 1 summarizes the lattice spacings of the seven most intense diffraction spots in the SAED pattern of Figure 3c, which are indexed to the crystalline Mn­(OH)_2_ phase with the space group *P*3̅*m*. Lattice spacing measurements were calibrated using Au nanoparticles, yielding a standard deviation of 0.2 Å. These findings suggest that the Mn­(OH)_2_ phase in the nanosheets is no longer single crystalline; instead, variations in zone axes between neighboring Mn­(OH)_2_ layers may give rise to the observed complexity in electron diffraction spots. Despite their air stability, the nanosheets were highly sensitive to the electron beam. High-resolution STEM imaging was unsuccessful since the structure rapidly degraded upon scanning. In contrast, the TEM mode enabled the acquisition of high-resolution images, although prolonged exposure still induced structural disruption. Figure S6 illustrates a metastable structural transition observed during repeated TEM imaging. Ultimately, the crystalline structure of the core–shell nanosheets was destroyed, as confirmed by the evolution of the HRTEM image after repeated TEM scanning. In addition to the dominant nanosheets, minor Te nanoparticles with coral-like morphology were identified, which is confirmed by STEM-EDS and SAED (Figure S7). These particles were much smaller than the nanosheets. Elemental mapping of nanosheets by STEM–EDS (Figure 3d–f) confirms the homogeneous distribution of Mn, Te, and O. STEM-EDS analyses on 10 nanosheets (Table 2) reveal compositional variations among these nanosheets, with Mn ranging from 23.3 to 28.8 at. % and Te from 14.2 to 20.6 at. %.

**Figure 3:**
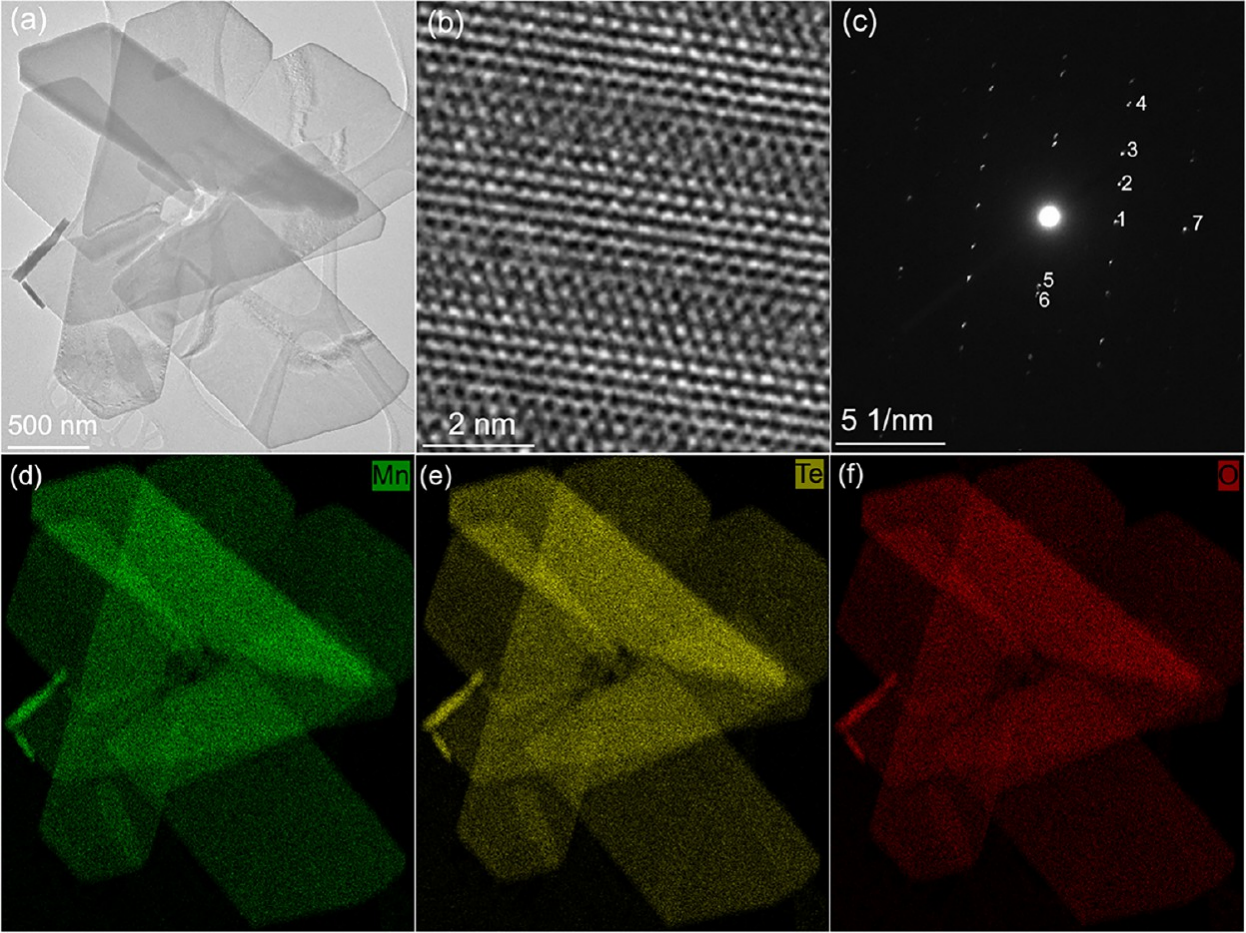
Plan-view TEM characterization of chemically exfoliated Mn–Te–O core–shell nanosheets. (a) Bright-field TEM image of Mn–Te–O nanosheets after dilution of the initial suspension. (b) HRTEM image of an individual nanosheet. (c) SAED pattern of a Mn–Te–O nanosheet. (d–f) Elemental mapping of the exfoliated flakes shown in panel (a), corresponding to (d) Mn, (e) Te, and (f) O distributions.

**Table 1:** Lattice Spacings from the SAED Pattern of One Nanosheet

diffraction spot	1	2	3	4	5	6	7
1/*d* (1/nm)	3.378	3.905	4.772	6.644	3.339	3.738	6.648
*d* (Å)	2.960	2.561	2.096	1.505	2.995	2.676	1.504
Miller indices (*hkl*) from Mn(OH)_2_ ^27^	(100)	(011)	(102)	(003)	(100)	(101)	(111)
simu. *d* from Mn(OH)_2_ ^27^ (Å)	2.877	2.459	1.828	1.578	2.877	2.459	1.567

**Table 2:** Plan-View STEM-EDS Composition of 10 Nanosheets

sample #	1	2	3	4	5	6	7
Mn (at.%)	27.6(1.1)	23.9(4)	26.0(2)	27.2(9)	25.2(1.1)	28.8(2)	27.9(1)
Te (at.%)	16.9(1.1)	14.2(5)	15.9(5)	17.3(1.0)	16.0(9)	16.8(2)	15.8(3)
O (at.%)	55.5(1.9)	61.8(6)	58.2(3)	55.4(9)	58.7(1.1)	54.3(6)	56.3(6)
sample #	8	9	10				
Mn (at.%)	27.6(8)	25.2(2)	23.3(1.1)				
Te (at.%)	19.2(8)	16.8(2)	20.6(6)				
O (at.%)	53.2(2.2)	58.0(9)	56.0(1.5)				

AFM measurement shows that the nanosheet has a thickness of ∼23 nm. (Figure 4a,b). To further validate the thickness, we conducted EELS-based plasmon spectroscopy. Figure 4c–f presents a representative TEM-EELS characterization of a core–shell nanosheet. The thickness mapping based on the mean free path (Figure 4d) confirms a uniform thickness in the nanosheet. The energy-loss spectrum exhibits a pronounced plasmon peak near 20 eV, corresponding to a thickness of ∼25 nm (Figure 4e). Furthermore, the Mn-M edge was clearly detected with energy-selective slit acquisition (Figure 4f), confirming a homogeneous Mn distribution in the core–shell nanosheets. A slight discrepancy is observed between the nanosheet thicknesses measured by AFM and EELS. AFM measurements are performed on Si/SiO_2_ substrates, which can adsorb a thin layer of solvent or ambient moisture and thereby influence the nanosheet height. In addition, AFM measurements typically carry an uncertainty of ± 0.5 nm. In contrast, EELS measurements are conducted on suspended nanosheets on TEM grids and generally have a relative uncertainty of ± 5–10%. The small difference in thickness observed between AFM and EELS falls well within the expected measurement uncertainty of the two techniques.

**Figure 4:**
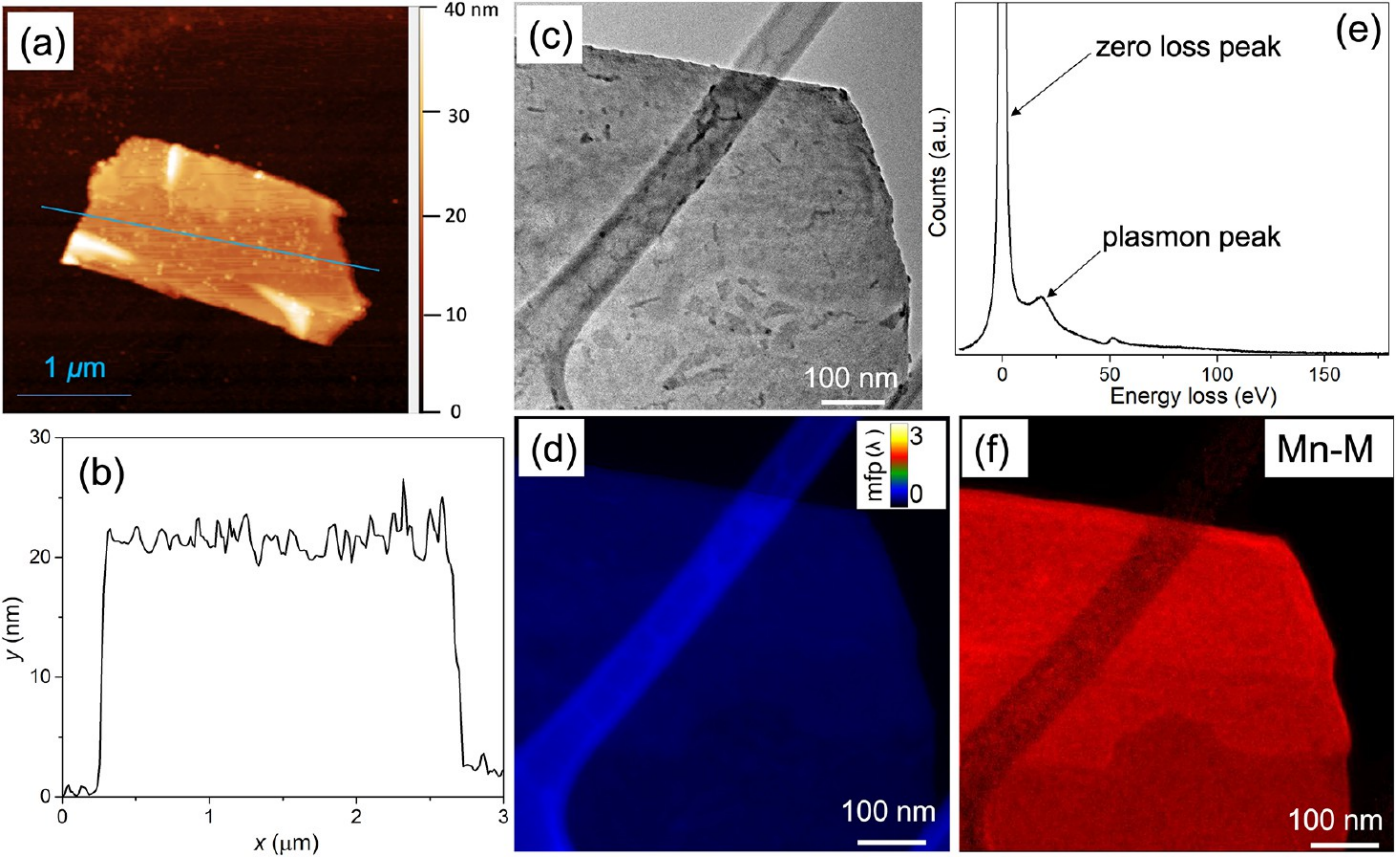
Characterization of a core–shell nanosheet by using AFM and EELS. (a) AFM mapping of a core–shell nanosheet. (b) Thickness profile along the direction highlighted in panel (a). (c) TEM image of a single Mn–Te–O nanosheet. (d) Mean free path map illustrating thickness variations. (e) EELS spectrum showing plasmon-loss peak of the nanosheet in panel (c). (f) Energy-loss intensities of Mn-M electrons.

The nanosheets are generally thicker than 20 nm. To investigate the origin of this relatively large thickness, cross-sectional analysis was performed on an FIB-cut lamella prepared from the restacked core–shell nanosheet pellet. Figure 5a shows side-view TEM images of all restacked nanosheets obtained via the FIB-cut. Figure 5b, c presents zoomed-in HRSTEM images, clearly revealing a core–shell architecture. Additionally, the core is crystalline while the shell is amorphous. Figure 5d, e shows elemental distributions of Mn and Te. The elemental distributions of Mn and Te are well separated. While the core region contains Mn and no Te, the shell region contains Te but no Mn. Since these core–shell nanosheets were prepared in Milli-Q water in air, it is possible that the Te shell absorbed some oxygen or underwent oxidation. To verify the oxygen distribution within the Te shell, we performed STEM–EDS mapping to highlight the spatial distributions of Te and O. Figure 5f reveals that the amorphous shell consists of Te and O, which indicates either oxygen adsorption at the surface or the formation of amorphous Te oxides. Together with Rietveld PXRD refinement and plan-view TEM results, this confirms that the nanosheets are composed of crystalline Mn­(OH)_2_ cores encapsulated by amorphous Te–O shells. Notably, the amorphous Te–O forms a shell exceeding 10 nm in thickness surrounding the Mn­(OH)_2_ cores, providing effective protection to highly air-sensitive Mn­(OH)_2_ against oxidation in water under ambient conditions. The reaction time seems to play an important role in the thickness of the Te–O shell. The shell originates from the growth of Te–O on crystalline Mn­(OH)_2_. Since the entire process of forming the core–shell nanosheets takes approximately 10 min, all nanosheets exhibit a relatively uniform Te–O shell thickness. Inhomogeneity is evident in the shell region, as shown in Figure 5c, a feature also observed for other core–shell nanoparticles.^31,45–49^ This inhomogeneity is likely linked to defect formation at the core–shell interface, which arises from the large lattice mismatch between the crystalline Mn­(OH)_2_ core and amorphous Te–O shell. Although elemental Te itself is not air-sensitive, the presence of defects can significantly influence local oxygen incorporation during spontaneous shell formation. Regions with a higher density of defects tend to accommodate more oxygen, leading to spatial variations in the Te/O ratio across the shell. These defect-mediated variations can produce oxygen-rich domains so that the shell in the high-resolution cross-sectional STEM–EDS images appears to have different regions rather than a fully homogeneous Te–O distribution. The apparent layering reflects local structural and compositional heterogeneities induced by defect sites rather than a uniform layer-by-layer growth process.

**Figure 5:**
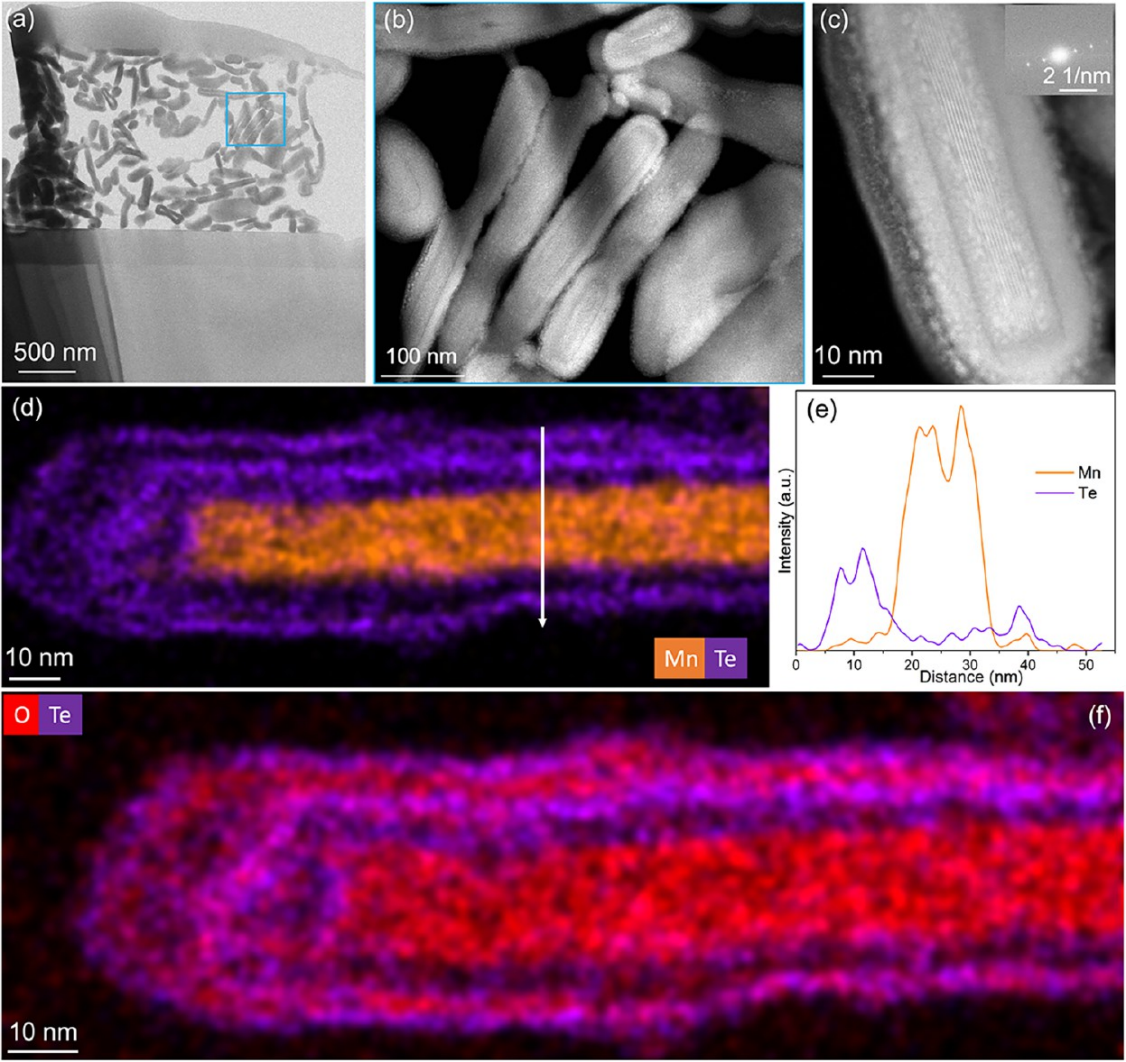
Cross-sectional imaging and compositional characterizations of restacked core–shell nanosheets. (a) Bright-field cross-sectional STEM image of a FIB-prepared lamella. (b) Magnified high-angle annular dark-field (HAADF) STEM image from the blue-highlighted region in panel (a), revealing the core–shell structure. (c) HAADF STEM image of a single nanosheet; the inset shows the corresponding fast Fourier transform (FFT) pattern. (d) Cross-sectional STEM–EDS elemental mapping showing the distributions of Mn and Te. (e) Elemental intensity profile showing the distribution across the nanosheet. (f) Cross-sectional STEM–EDS elemental maps highlighting the spatial distribution of Te and O.

Based on the overall reaction shown in Figure 1, we speculate that Te^2–^ ions are expelled from the Mn framework in association with Li deintercalation. Once in solution, a portion of the Te^2–^ species is oxidized by protons (or possibly by dissolved oxygen) to form elemental Te, while others may undergo concurrent hydrolysis and oxidation to yield TeO_*x*_. Owing to the rapid kinetics and the insolubility of TeO_*x*_, these species precipitate near the Mn­(OH)_2_ surface, forming a conformal shell rather than diffusing away. In contrast, elemental Te formed alongside gaseous byproducts (H_2_) is more likely to disperse into the solution rather than remain adhered to the Mn­(OH)_2_ surface. Regarding the sequence of Te and TeO_*x*_ formation, multiple pathways are plausible. One possibility is that TeO_*x*_ forms first by consuming local water (primarily OH^–^) or dissolved O_2_, leaving subsequently expelled Te^2–^ in solution to be protonated to form TeH_2_, which then decomposes into H_2_ and elemental Te. Alternatively, Te may form initially through the generation and decomposition of TeH_2_, which would produce OH^–^ as a byproduct and render the solution more basic, thereby favoring the subsequent formation of TeO_*x*_. Regardless of the sequence, we suspect that both TeO_*x*_ and elemental Te originate from reactions between Te^2–^ and OH^–^/H^+^ species derived from water, a process that is also coupled with the hydrolysis of Mn. Overall, we believe that several factors, including the deintercalation process, redox events, and solution pH, likely contribute to the spontaneous formation of the Te–O shell. Fully understanding the mechanism will require extensive control experiments and advanced characterizations, which are beyond the scope of this study but represent interesting directions for future investigation.

Additional side-view HRSTEM images (Figure S8) reveal that different nanosheets contain Mn­(OH)_2_ cores ranging from 7 to 20 layers, which accounts for the compositional variations observed among individual core–shell nanosheets in the plan-view STEM-EDS results. The FFT pattern (inset of Figure 5c) indicates imperfect nanosheet alignment, as sharp diffraction spots were observed only along one direction, preventing the determination of the zone axis by symmetry. The lattice spacing extracted from the FFT pattern is ∼9.0 Å, consistent with the PXRD peak at 4.4° of 2θ. Such a lattice spacing is much larger than the maximum interlayer spacing of *P*3̅*m* Mn­(OH)_2_ (4.73 Å). This expansion may be attributed to the strong hydration of Mn­(OH)_2_, a common feature of layered compounds in aqueous solution.^50,51^

### Magnetic Properties

After obtaining a large quantity of the core–shell nanosheets, we investigated their magnetic properties using 3.3 mg of restacked material. Hydration effects and the presence of minor impurity phases introduced large uncertainties in the molecular weight, making it difficult to reliably determine the effective magnetic moment based on Curie–Weiss fitting. However, the impurity phases are mostly diamagnetic; therefore, we performed temperature- and field-dependent magnetization to test the magnetic properties of Mn­(OH)_2_ nanosheets (Figure 6). The temperature-dependent magnetization of the restacked pellet reveals a clear antiferromagnetic transition at 11 K (Figure 6a), observed under applied magnetic fields of 1000, 5000, and 10000 Oe. No divergence was detected between the zero-field-cooled (ZFC) and field-cooled (FC) curves. The inverse susceptibility of the 5000 Oe ZFC *M–T* data is linear above 11 K (Figure 6b). The linearity of the inverse susceptibility above 11 K indicates Curie–Weiss paramagnetic behavior, while the deviation below this temperature suggests a magnetic phase transition, consistent with the onset of long-range magnetic ordering around 11 K. Field-dependent magnetization was measured at 1.8, 100, and 300 K, with all three *M*–*H* curves exhibiting linear behavior. The linear *M*–*H* response at 1.8 K, below the antiferromagnetic (AFM) transition temperature, is characteristic of AFM ordering, whereas the *M*–*H* curves at 100 and 300 K reflect paramagnetic behavior (Figure 6c). These results identify the core–shell nanosheets as a 2D magnetic material with AFM ordering, providing a useful platform for fundamental studies of low-dimensional magnetism in air-sensitive samples.

**Figure 6:**
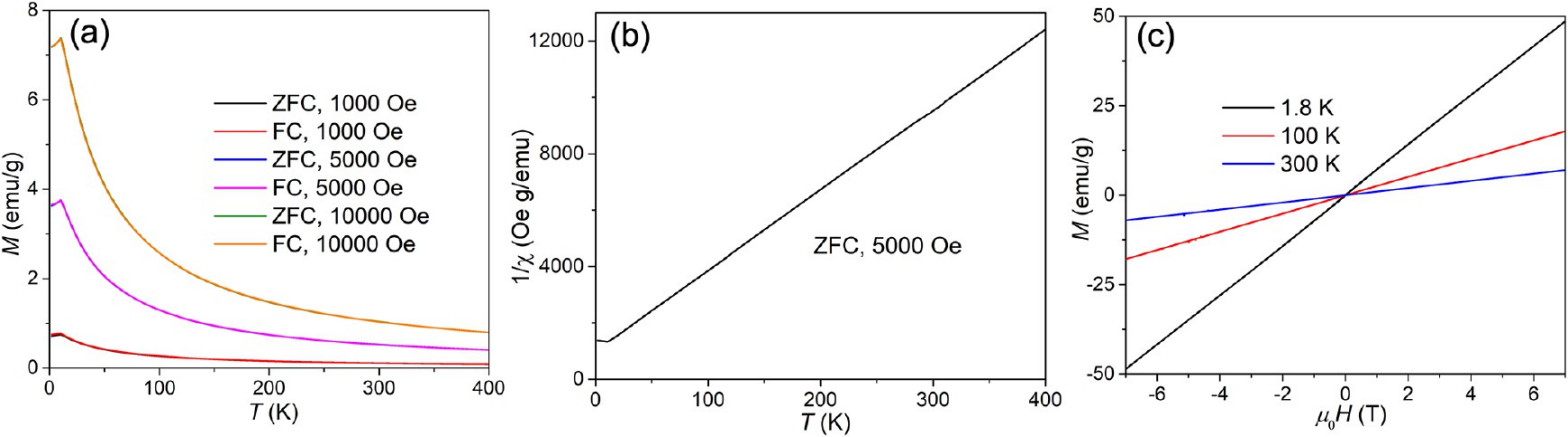
Temperature- and field-dependent magnetization analysis. (a) Temperature-dependent magnetization. (b) Inverse susceptibility under a magnetic field of 5000 Oe. (c) Field-dependent magnetization.

Typically, the effect of dimensionality on magnetic properties is evident, influencing both the type and temperature of magnetic ordering.^52–54^ For example, it is common that the ordering temperature decreases pronouncedly due to enhanced thermal fluctuations in thinner flakes. Consequently, the magnetic ordering of few-layer Mn­(OH)_2_ might differ from that of bulk Mn­(OH)_2_. However, our chemically synthesized core–shell nanosheets exhibit magnetic properties closely resembling those of the bulk phase, which shows an AFM ordering at 12 K.^27^ Additionally, reliable magnetic characterization of 2D materials, particularly those that are air-sensitive, typically requires encapsulation within air-stable, nonmagnetic films or measurement in an inert gas environment to minimize environmental and thermal perturbations.^53,55^ Despite containing a few-layer highly air-sensitive Mn­(OH)_2_, our core–shell nanosheets can be characterized without such protective measures. In the case of our core–shell nanosheets, we hypothesize that the amorphous Te–O shell in our system naturally encapsulates and protects the few-layer Mn­(OH)_2_ cores, thereby suppressing the influence of external disturbances and eliminating the need to protect air-sensitive Mn­(OH)_2_. Overall, the Te–O shell stabilizes the crystallinity and preserves the antiferromagnetic order even at few-layer thicknesses.

### Potential Electronic Applications

The printability of the core–shell nanosheet suspension was evaluated on various substrates, demonstrating that it can be uniformly drop-cast on substrates such as indium tin oxide (ITO)-coated glass, PET, and a Si/SiO_2_ wafer. As shown in Figure 7, the deposited films exhibit good coverage on the substrates, including the edge regions. The films are composed of packed core–shell nanosheets, consistent with the morphology observed in our TEM analyses (Figures 3, S2, S3, and S5). To determine the film thickness, we attempted to prepare cross-sectional samples by using FIB cutting. However, FIB cutting could not be applied to the film on PET because PET has a low melting point (approximately 150 °C) and was damaged by the ion beam. In contrast, the cross-sectional images of the films on ITO-coated glass and Si/SiO_2_ wafer show relatively uniform thicknesses. Additionally, the side-view SEM images further reveal that the deposited films on Si/SiO_2_ wafers with prepatterned electrodes have a low density and discontinuous microstructure, which likely contributes to a higher resistivity. To investigate the electronic transport properties, the deposited film on a silicon wafer with prepatterned electrodes fabricated by photolithography was measured (Figure 7c). Our transport measurement shows that the core–shell nanosheets deposited on the Si/SiO_2_ wafer have resistance values at least in the megaohm range at room temperature. These findings suggest that optimization of the printing process and device architecture may hold promise for electronic devices, particularly in capacitors and transistors requiring ultrathin insulating layers for safety reasons. Additionally, the insulating nature of the core–shell nanosheets points to possible optoelectronic applications.

**Figure 7:**
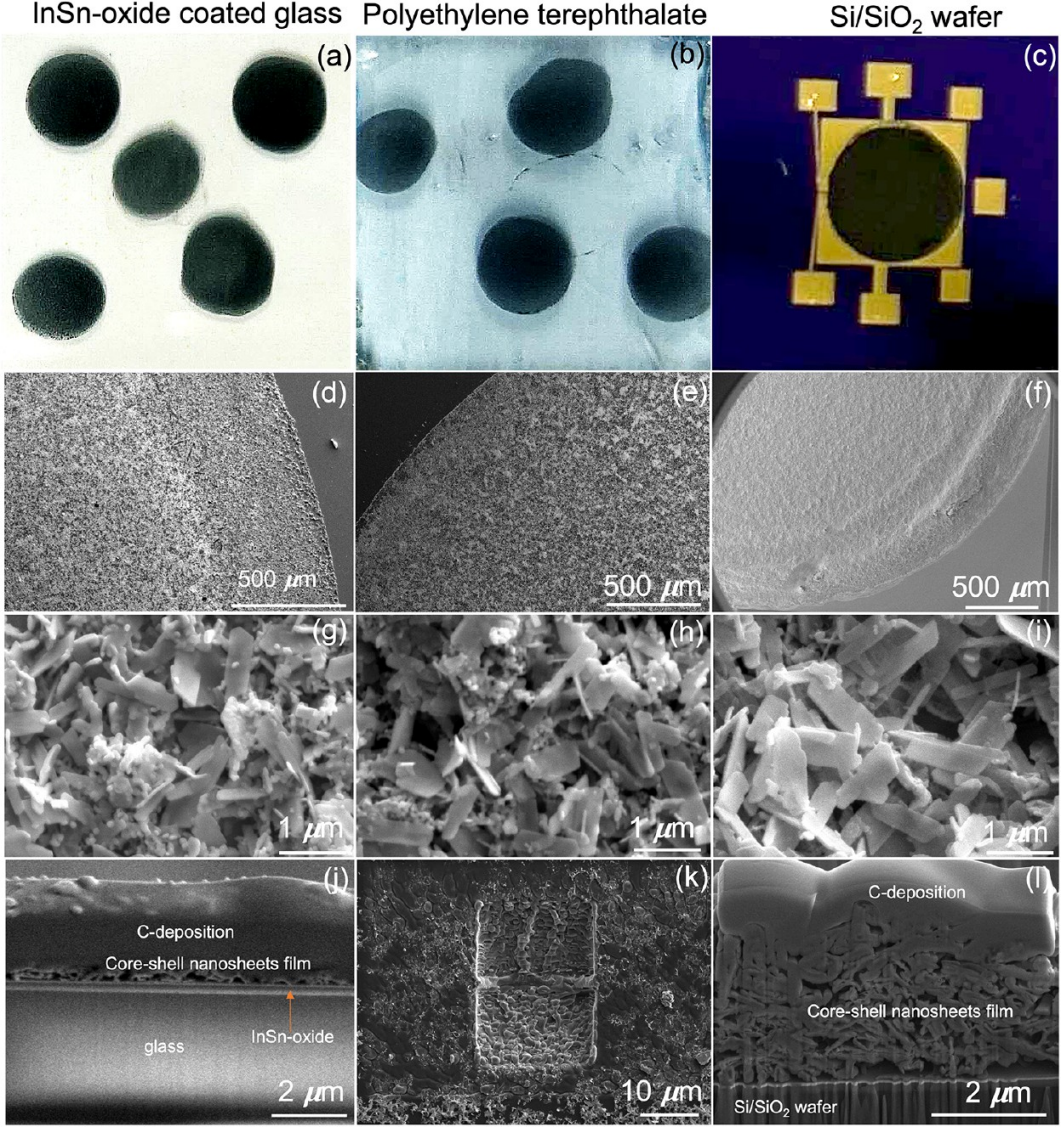
Characterization of the films deposited on different substrates. Optical images, low-magnification SEM images, high-magnification SEM images, and cross-sectional SEM images of core–shell nanosheet films on ITO-coated glass (a, d, g, j), PET film (b, e, h, k), and Si/SiO_2_ wafers with prepatterned electrodes (c, f, i, l).

To better assess the charge transport and underlying conductivity mechanism, we prepared pressed pellets for resistivity measurements. The temperature-dependent resistivity of the pellet (Figure 8) shows an insulating behavior. Arrhenius fitting in the 150–300 K temperature range yields an activation energy of approximately 0.08 eV, corresponding to a transport gap of ∼0.16 eV. At lower temperatures, the rapidly increasing resistivity leads to enhanced Joule heating, which may cause deviations from linear Arrhenius behavior. Such deviation may also arise from the presence of metallic Te impurities and/or hopping conduction between core–shell nanosheets. Overall, these additional data provide a more reliable understanding of the core–shell nanosheets’ electrical transport properties.

**Figure 8:**
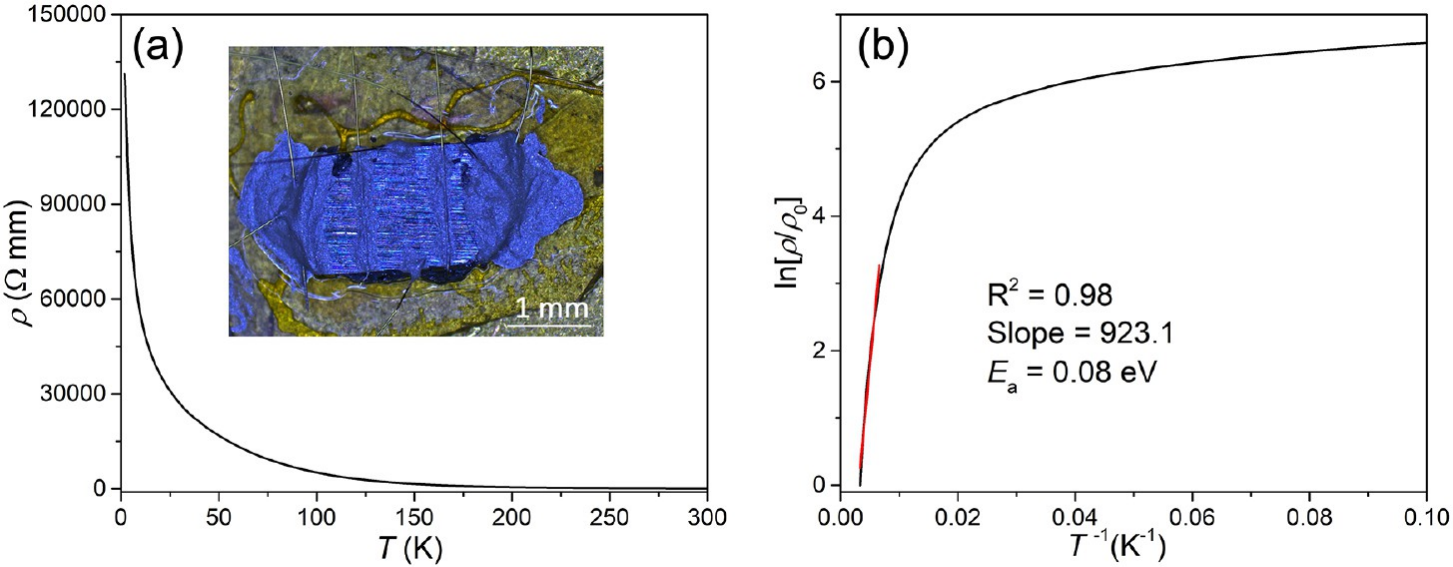
Transport properties of the pressed pellet. Temperature-dependent resistivity (a) and natural logarithm (b) of the relative resistivity as a function of the inverse absolute temperature. The red line represents the linear Arrhenius fit performed over the range of 150–300 K. The inset shows the device configuration used for the four-probe transport measurement.

## Conclusions and Outlook

In conclusion, we present a simple one-step route for the preparation of a core–shell nanomaterial. The material features a predominantly amorphous Te–O shell that effectively protects a few-layer Mn­(OH)_2_ core. Therefore, these core–shell nanosheets are stable for at least 31 days under ambient conditions. Magnetic measurements reveal an antiferromagnetic transition at 11 K, consistent with that of bulk Mn­(OH)_2_. These results demonstrate that the magnetic properties of bulk Mn­(OH)_2_ are retained in few-layer nanosheets. We also explored the potential of this material for printable electronics. Suspension of the core–shell nanosheets can be easily drop-cast on PET, ITO-coated glass, and Si/SiO_2_ wafers, forming uniform circular patterns with resistance values in the megaohm range at least. This printability highlights their promise for device applications that require thin insulating layers.

The findings reported here advance the development of 2D materials derived from highly air-sensitive quasi-layered bulk compounds. We demonstrate how a simple chemical exfoliation protocol leads to the formation of stable core–shell nanosheets. The unique combination of distinctive magnetic behavior, air stability, and printability might open opportunities for technological applications and future research. Remarkably, the synthesis requires only 10 min of sonication in Milli-Q water under ambient conditions. Overall, this work underscores the effectiveness of chemical exfoliation as a powerful approach for producing novel core–shell nanomaterials. Looking ahead, future efforts to refine exfoliation strategies will enable the synthesis of more uniform core–shell nanosheets while preserving their bulklike physical properties, thereby advancing both fundamental research and real-world technological applications.

## Supplementary Material



## Notes

ICP-OES results of parent Li_1+*x*_MnTe_2_, core–shell nanosheets, and MeOH-washed Li_1+*x*_MnTe_2_; exfoliation test of Li_1+*x*_MnTe_2_ in different solvents; TEM images of overlapping nanosheets and a diluted nanosheet suspension; structural evolution of core–shell nanosheets under 34 days of air exposure and prolonged electron beam exposure; TEM/STEM images and SAED patterns of diverse core–shell nanosheets; Te coral-like nanoparticles characterization; additional STEM images of core–shell nanosheets obtained from a FIB-cut lamella (PDF)

## ○

Department of Chemistry, Columbia University, New York, NY 10027, USA
