# Supplementary material for: Printable and Antiferromagnetic Mn(OH)2@Te–O Core–Shell Nanosheets
Source: Chem Mater. 2026 Jan 27;38(3):1274–86. doi: 10.1021/acs.chemmater.5c02655 (PMC12895390; doi:10.1021/acs.chemmater.5c02655)
Supplement: Supplementary file 1 [file cm5c02655_si_001.pdf]

Supporting Information: Printable and Antiferromagnetic $Mn(OH)_2$@Te-O Core-shell Nanosheets

Fang Yuan,[†] Jiaze Xie,[†] Ratnadwip Singha,[‡] Christie S. Koay,[†] Sigalit Aharon,[†] Guangming Cheng,[¶] Brianna L. Hoff,[†] Vojtech Kundrat,[§] Xiaoyu Song,[‖] Sudipta Chatterjee,[†] Lothar Houben,[⊥] Jakub Zalesak,[#] Nan Yao,[¶] and Leslie M. Schoop[*,†]

†*Department of Chemistry, Princeton University, Princeton, New Jersey 08544, USA*

‡*Department of Physics, Indian Institute of Technology Guwahati, Assam 781039, India*

¶*Princeton Materials Institute, Princeton University, Princeton, New Jersey 08544, USA*

§*Department of Chemistry, Faculty of Science, Masaryk University, Kamenice 5, Brno 62500, Czechia*

‖*Department of Chemistry, Princeton University, Princeton, New Jersey 08544, USA*

Present address: Department of Chemistry, Columbia University, New York, NY 10027, USA

⊥*Department of Chemical Research Support, Weizmann Institute of Science, Rehovot 7610001, Israel*

#*Chemistry and Physics of Materials, University of Salzburg, 5020 Salzburg, Austria*

E-mail: lschoop@princeton.edu

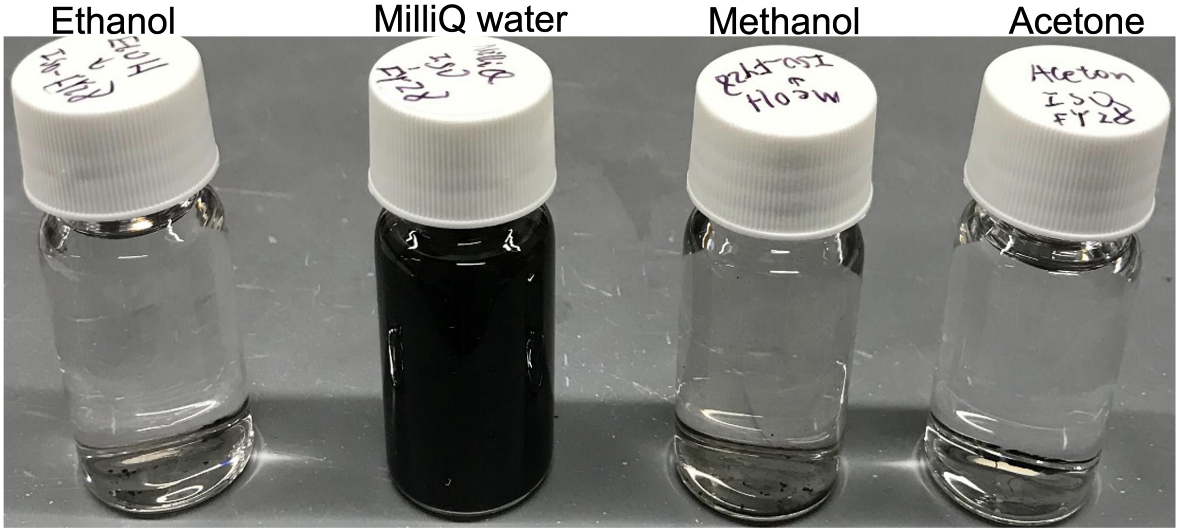

Figure S1: Exfoliation comparison of $Li_{1+x}MnTe_2$ in different solvents. From left to right: ethanol, Milli-Q water, methanol, and acetone.

Table S1: ICP-OES results of parent $Li_{1+x}MnTe_2$, core-shell nanosheets, and MeOH-washed $Li_{1+x}MnTe_2$

	Li (ppm)	Li content in Li_xMnTe_2
Parent $Li_{1+x}MnTe_2$ 1#	3.8	1.8
Parent $Li_{1+x}MnTe_2$ 2#	4.1	1.9
Parent $Li_{1+x}MnTe_2$ 3#	4.3	2.0
Core-shell nanosheets 1#	N/A	N/A
Core-shell nanosheets 2#	N/A	N/A
Core-shell nanosheets 3#	N/A	N/A
MeOH-washed $Li_{1+x}MnTe_2$ 1# (large crystals)	2.3	1.0
MeOH-washed $Li_{1+x}MnTe_2$ 2# (crushed crystals)	0.3	0.1
MeOH-washed $Li_{1+x}MnTe_2$ 3# (crushed crystals)	0.5	0.2

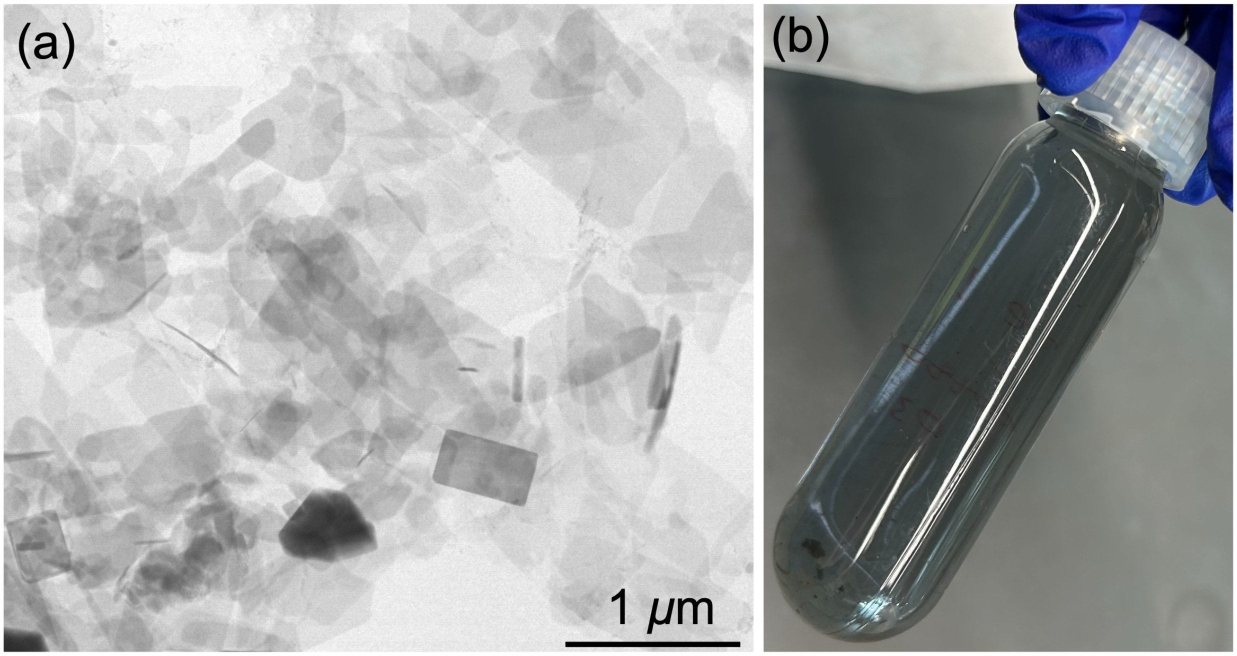

Figure S2: Sample preparation for plan-view S/TEM. (a) TEM image of the inital nanosheet suspension deposited on a Cu TEM grid. (b) Photograph of the nanosheet suspension after 20 times dilution.

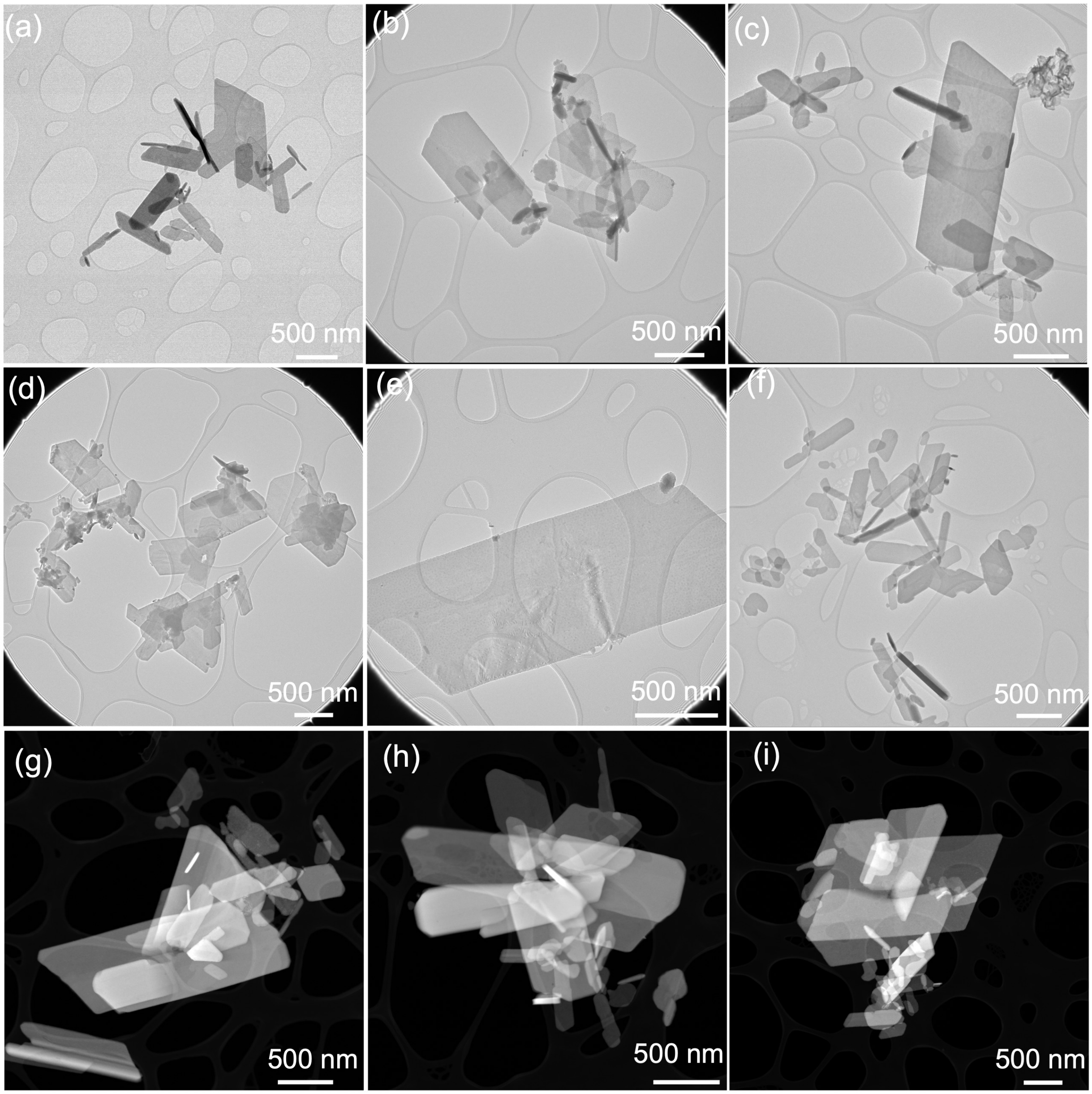

Figure S3: TEM/STEM images of diverse core-shell nanosheets.

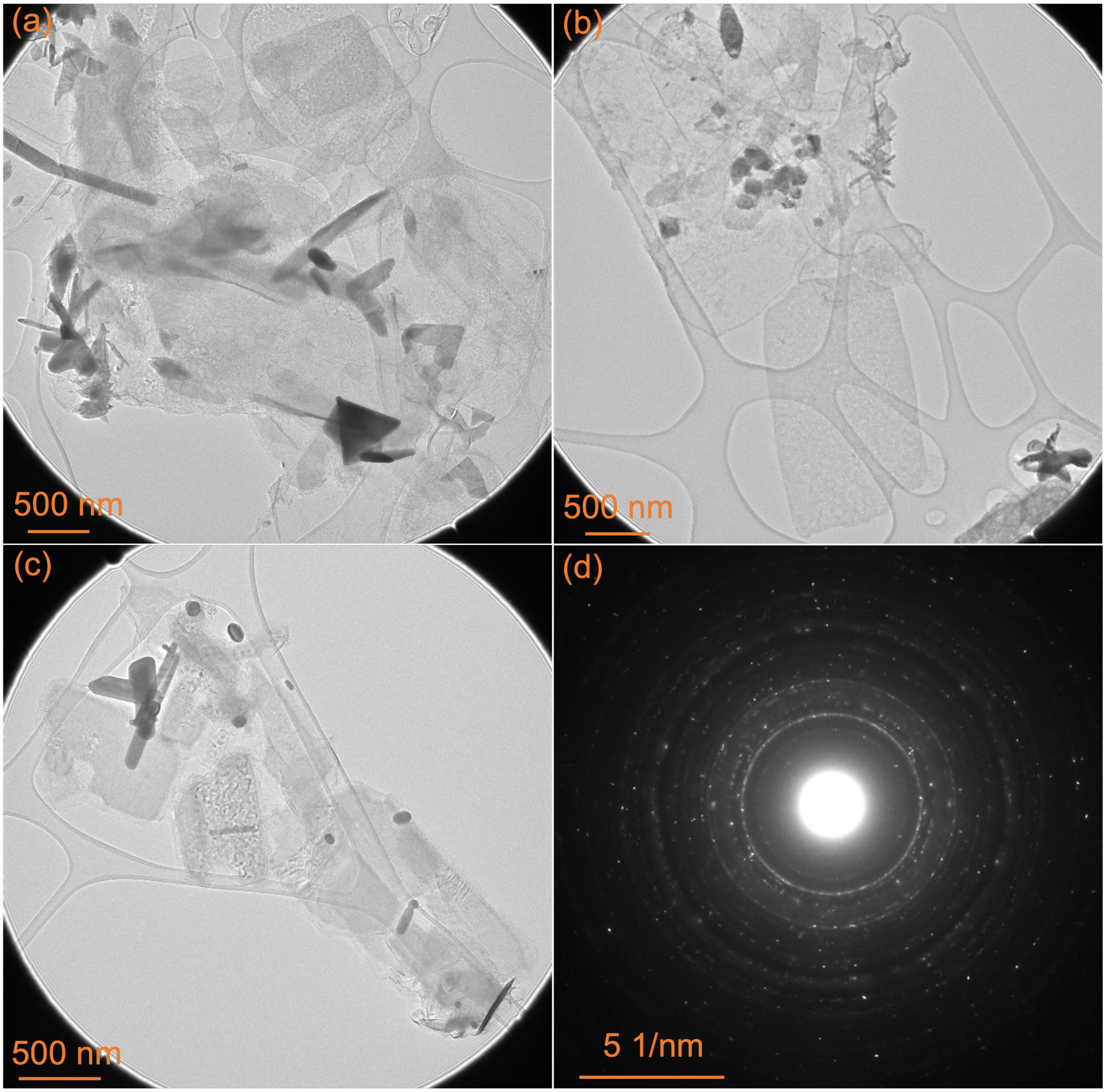

Figure S4: Degradation of core-shell nanosheets after 34 days in air. (a-c) TEM images. (d) SAED pattern.

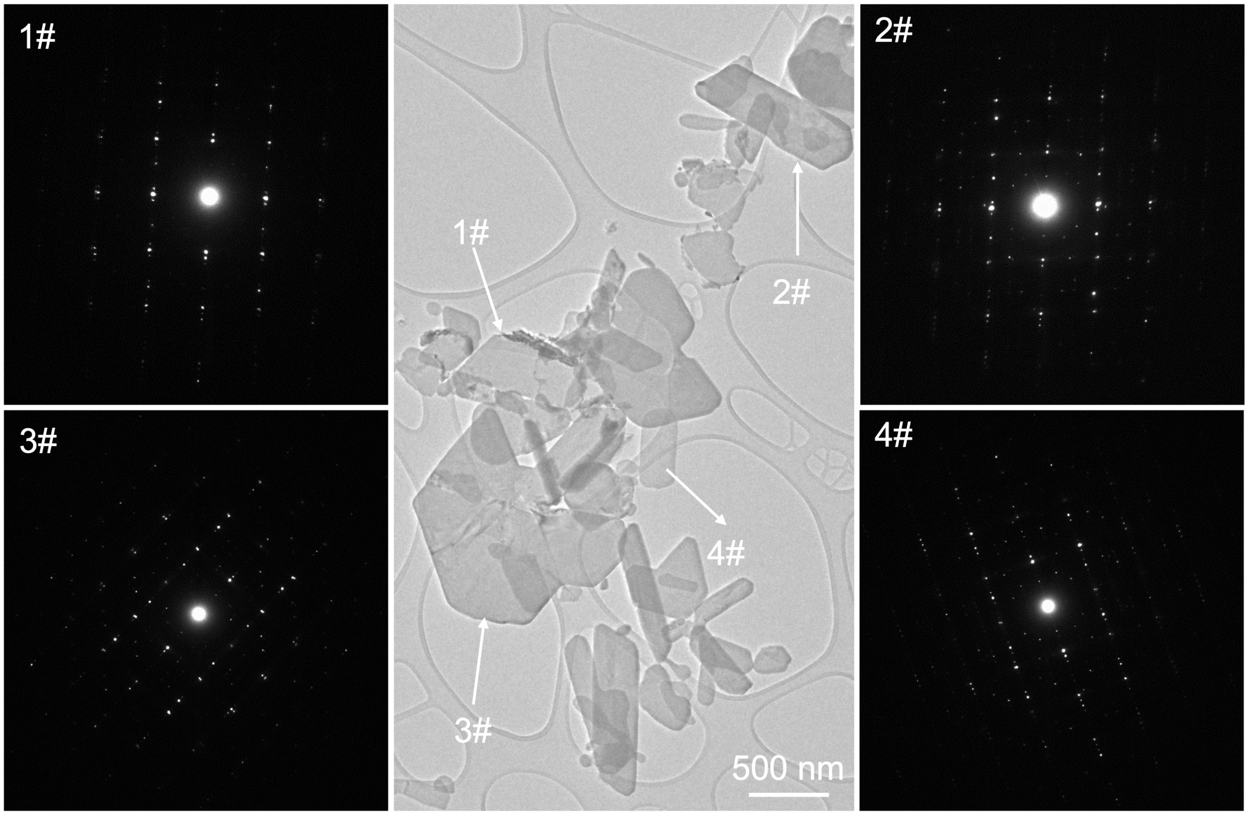

Figure S5: SAED patterns of various core-shell nanosheets.

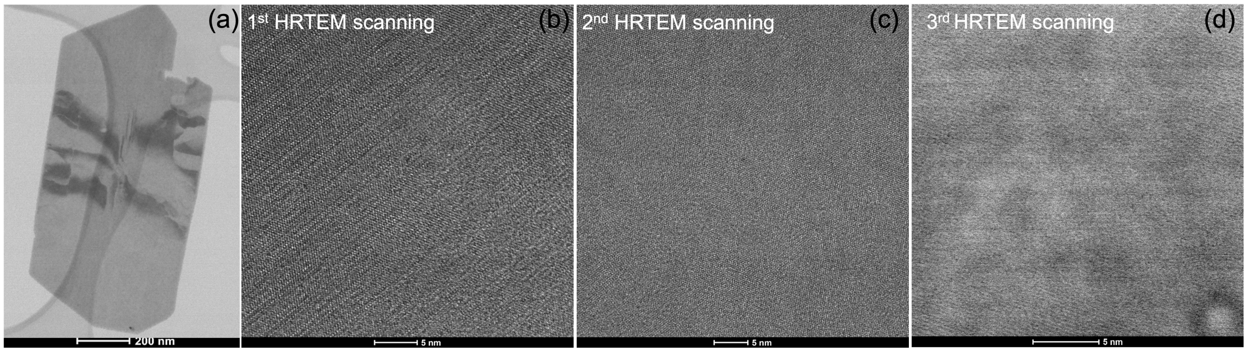

Figure S6: Repeated HRTEM imaging of core–shell nanosheets. (a) TEM image of a selected nanosheet. (b) HRTEM image from the first scan. (c) HRTEM image from the second scan (d) HRTEM image from the third scan.

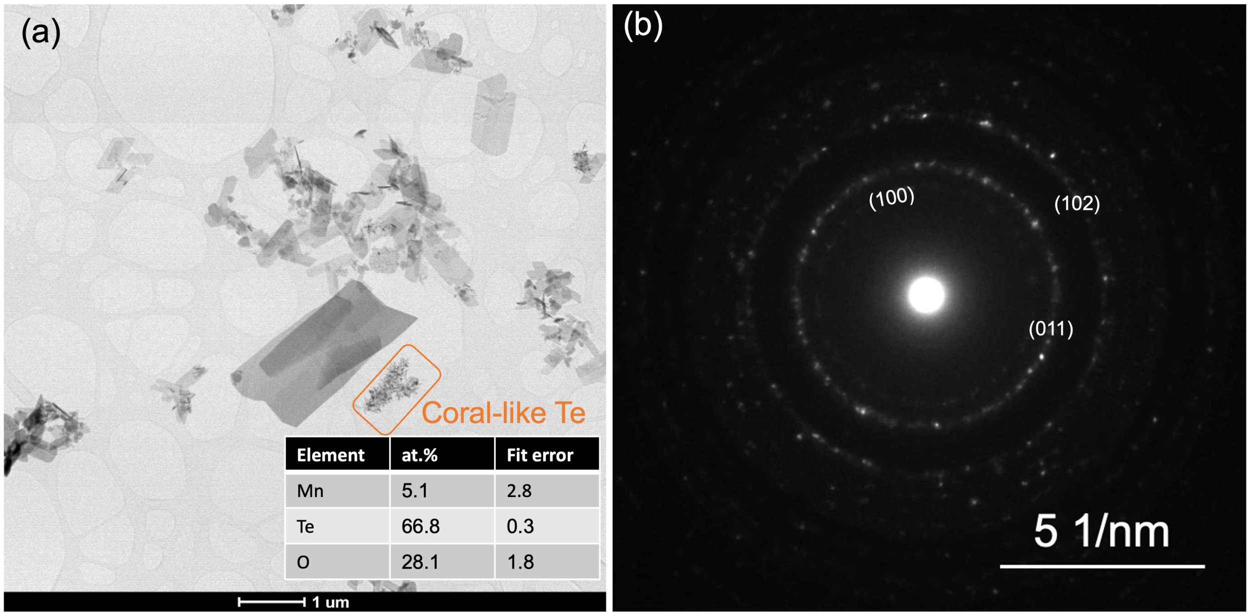

Figure S7: Te coral-like nanoparticles characterization. (a) TEM image and STEM-EDS results of Te nanoparticles. (b) SAED patterns of Te nanoparticles.

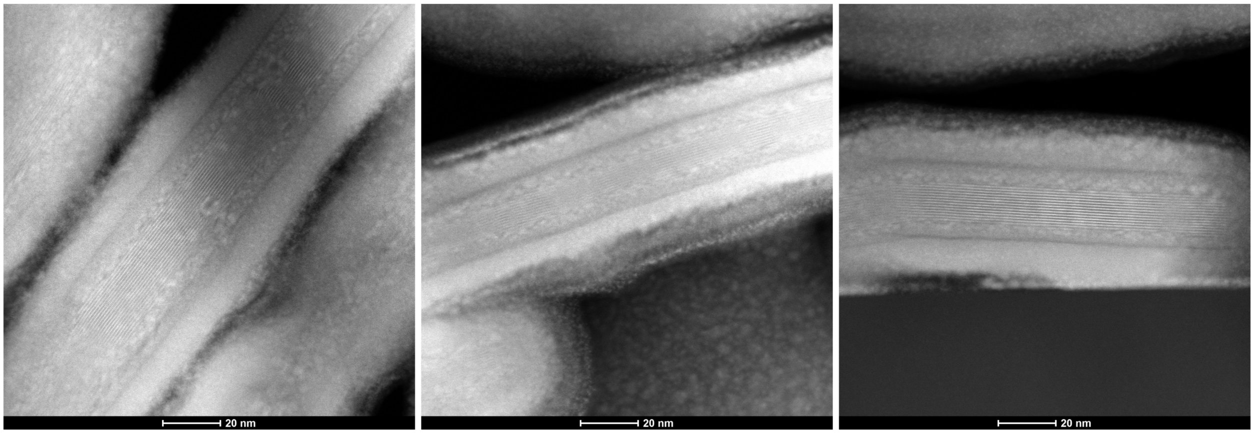

Figure S8: Cross-sectional HAADF STEM image of more core-shell nanosheets from FIB-cut lamella.
